# Exercise efficiency relates with mitochondrial content and function in older adults

**DOI:** 10.14814/phy2.12418

**Published:** 2015-06-09

**Authors:** Nicholas T Broskey, Andreas Boss, Elie-Jacques Fares, Chiara Greggio, Gerald Gremion, Leo Schlüter, Didier Hans, Roland Kreis, Chris Boesch, Francesca Amati

**Affiliations:** 1 Department of Physiology, School of Biology and Medicine, University of LausanneLausanne, Switzerland; 2 Department of Clinical Research & Institute of Interventional, Diagnostic and Pediatric Radiology, University of BernBern, Switzerland; 3 Sports Medicine Unit, University HospitalLausanne, Switzerland; 4 Service of Cardiology, University HospitalLausanne, Switzerland; 5 Center for Bone Disease, Bone & Joint Department, University HospitalLausanne, Switzerland

**Keywords:** Chronic exercise, delta efficiency, gross efficiency, net efficiency

## Abstract

Chronic aerobic exercise has been shown to increase exercise efficiency, thus allowing less energy expenditure for a similar amount of work. The extent to which skeletal muscle mitochondria play a role in this is not fully understood, particularly in an elderly population. The purpose of this study was to determine the relationship of exercise efficiency with mitochondrial content and function. We hypothesized that the greater the mitochondrial content and/or function, the greater would be the efficiencies. Thirty-eight sedentary (S, *n* = 23, 10F/13M) or athletic (A, *n* = 15, 6F/9M) older adults (66.8 ± 0.8 years) participated in this cross sectional study. 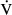
O_2peak_ was measured with a cycle ergometer graded exercise protocol (GXT). Gross efficiency (GE, %) and net efficiency (NE, %) were estimated during a 1-h submaximal test (55% 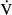
O_2peak_). Delta efficiency (DE, %) was calculated from the GXT. Mitochondrial function was measured as ATP_max_ (mmol/L/s) during a PCr recovery protocol with ^31^P-MR spectroscopy. Muscle biopsies were acquired for determination of mitochondrial volume density (MitoVd, %). Efficiencies were 17% (GE), 14% (NE), and 16% (DE) higher in A than S. MitoVD was 29% higher in A and ATP_max_ was 24% higher in A than in S. All efficiencies positively correlated with both ATP_max_ and MitoVd. Chronically trained older individuals had greater mitochondrial content and function, as well as greater exercise efficiencies. GE, NE, and DE were related to both mitochondrial content and function. This suggests a possible role of mitochondria in improving exercise efficiency in elderly athletic populations and allowing conservation of energy at moderate workloads.

## Introduction

Elderly adults typically display low levels of exercise efficiency, which could hamper normal activities of daily living (Martin et al. 1992; Woo et al. 2006). High levels of efficiency are important since they allow one to expend less energy for a given amount of physical work. Skeletal muscle mitochondrial dysfunction is suggested to lead to metabolic perturbations, particularly in older adults (Conley et al. 2000; Menshikova et al. 2006). These perturbations lead to changes with cellular dynamics and are associated with loss of muscle quality as well as increases in oxidative stress (Peterson et al. 2012). More recently, Santanasto et al. (2014) also described this relationship between mitochondrial dysfunction and muscle ailments by showing that a lower mitochondrial function relates to greater fatigability in older adults. A sedentary lifestyle is touted as the primary culprit for decreases in mitochondrial function and content (Broskey et al. 2014). On the other hand, physical activity, in the form of structured aerobic exercise, can help ameliorate these conditions (Lanza et al. 2008; Conley et al. 2013a).

Exercise efficiency is broadly defined as the ratio of mechanical work rate over energy expenditure and is typically expressed by four different equations with the most common being either gross or delta efficiency (Ettema and Loras 2009). Historically, Gaesser and Brooks (1975) described the latter as being the most preferred and reliable measure at moderate and high power outputs. Comparisons of exercise efficiency between young active versus young sedentary adults show that those who are active have a higher efficiency than those who are sedentary (Mogensen et al. 2006). Young and old comparisons show that older adults have decreases in efficiency and/or expend more energy for walking (Martin et al. 1992; Malatesta et al. 2003; Mian et al. 2006; Ortega and Farley 2007; Conley et al. 2013b). We have previously shown that older obese sedentary adults improved exercise efficiency with 4 months of exercise alone or in combination with weight loss (Amati et al. 2008), however it is still uncertain how exercise efficiency differs between older adults who are habitually active versus age-matched sedentary counterparts.

Furthermore, the relationship between this difference in efficiency and the extent at which mitochondrial content and function contribute to these differences has only been partially explored. A couple of groups already showed in young adults that no relationship exists between exercise efficiency and mitochondrial content measured by citrate synthase activity (Mallory et al. 2002; Mogensen et al. 2006). Conley et al. (2013b) showed that reduced mitochondrial efficiency correlates with reduced delta efficiency; however, this was in a young and old cross-sectional comparison.

The purpose of this paper was to look at differences in exercise efficiency between older athletic adults and older sedentary adults matched for age. We hypothesized that athletic older adults will have higher exercise efficiencies as well as higher mitochondrial content and function. A secondary hypothesis was that this increased exercise efficiency related to increased mitochondrial content and function.

## Methods

### Subjects

Older adults between the age of 60 and 80 years old in good general health, nonsmokers, and weight stable were recruited for this study. Volunteers were considered athletes or sedentary based on self-reported frequency of habitual levels of exercise. Those who are considered athletes engaged in three or more structured aerobic exercise sessions per week for more than 1 year. Individuals were defined as sedentary if they engaged in one or less structured exercise sessions per week. Volunteers were excluded if they took any medication known to affect muscle metabolism, such as corticosteroids or enhancers of insulin sensitivity. The research protocol was accepted by the ethics committee of the Canton of Vaud and all volunteers gave written informed consent.

### 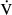
O_2peak_

Peak oxygen consumption was determined as described previously (Broskey et al. 2014). Briefly, the graded exercise test was conducted on an electronically braked cycle ergometer (Lode B.V., Groningen, The Netherlands) combined with continuous ECG, heart rate, and blood pressure recordings. 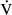
O_2_ was computed with indirect calorimetry (Metalyzer3B, Cortex GmbH, Leipzig, Germany). The exact protocol was the following with increments derived every 2 min: sedentary men started at 50 W and increased in increments of 25 W, male athletes started at 50 W and increased one increment of 50 W, increased again of 50 W, then the remainder were increments of 25 W, and all women started at 25 W and increased in increments of 25 W. Both groups performed the test until volitional exhaustion or if one of the American College of Sports Medicine established criteria for maximal testing had been reached (Walter et al. 2010).

### Submaximal bike test

Subjects were instructed to avoid strenuous exercise 2 days before the test. To ensure adequate glycogen stores, subjects were asked to eat their habitual diet ensuring that they eat at least 200 g of carbohydrates per day for the 3 days before the submaximal test as previously described (Amati et al. 2008). After a 12-h overnight fast, participants reported to the laboratory and biked for 1-h on the electronically braked cycle ergometer at the exact 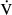
O_2_ corresponding to 55% of 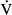
O2 peak. Subjects were instructed to pedal at a controlled cadence between 60 and 65 rpms due to this being reported previously as a stable cadence to calculate efficiency (Sidossis et al. 1992; Francescato et al. 1995). Oxygen consumption was recorded via indirect calorimetry for 5 min at four time points (15, 30, 45, and 60 min). This testing protocol was chosen to replicate what was published previously and was successful for outcomes of efficiency in sedentary and trained elderly individuals (Amati et al. 2008). Each volunteer was able to complete the 1-h test accordingly.

### DXA

Lean body mass (LBM) was determined by dual energy X-ray absorptiometry (DiscoveryA; Hologic Inc, Bedford, MA).

### Resting energy expenditure

On a separate day from the exercise testing, indirect calorimetry was used to determine resting energy expenditure (REE) using an open canopy system (Quark, Cosmed, Rome, Italy). After an overnight fast and stay at the Clinical Research Center at ∽6:30 A.M. subjects were placed under the canopy while resting in bed. They were told to close their eyes and try to sleep, refrain from fidgeting, and not to perform any type of activity (i.e., watching TV or reading). The duration was at least 30 min with the first 5 min of data discarded to assure steady state.

### Muscle biopsies

Percutaneous muscle biopsies were obtained following the REE, in the fasted state, from the *vastus lateralis* under local anaesthesia (buffered lidocaïne) as previously described (Amati et al. 2011). Controlled conditions included no exercise for 48 h, a standardized dinner (consisting of 7 kcal/kg of body weight with 50% carbohydrates, 20% proteins, and 30% fats) followed by an overnight fast prior to the biopsy. After trimming of visible adipose tissue with a dissecting microscope (MZ6; Leica Microsystems, Wetzlar, Germany), one portion of the specimen (5 mg) was fixed in glutaraldehyde solution (EMS, Hatfield, PA) for transmission electron microscopy as described previously (Broskey et al. 2013).

### Electron microscopy

Transmission electron microscopy was used to measure mitochondrial volume density (MitoVd) as a marker of mitochondrial content. A recent validation and detailed description of this stereological method has been described elsewhere (Broskey et al. 2013). Briefly, twenty micrographs of the intramyofibrillar region were taken per subject with a Philips CM100 transmission electron microscope (FEI, Eindhoven, The Netherlands) at an acceleration voltage of 80 kV and ×33,000 of magnification, with a pixel size of 2.11 nm and a horizontal field width of 8.3 *μ*m, with a Megaview III SIS digital camera (Olympus Soft Imaging Solutions, Münster, Germany), using the software SIS iTEM Megaview III (Olympus Soft Imaging Solutions). To avoid sampling bias, all images were taken using the Multiple Image Alignment plugin (MIA) of the iTEM software. The MIA plugin is a panorama-image function that allows acquisition of a larger image of the sample than the one observed by an automatic displacement of the sample under the electron beam. The micrographs are composed of an alignment of four images in the *x*-axis per five images in the *y*-axis, creating a large single micrograph of 20 images without changing the magnification. A grid was superimposed on each micrograph with squares of 500 × 500 (0.25 *μ*m^2^). Upon placing the grid, the number of points (defined as two intersecting grid lines) that touched the mitochondria, were tallied and divided by the total number of points on the grid. This process was repeated for the other 19 images and averaged together to receive the volume density percentage for each grid.

### PCr recovery

The rate of postexercise phosphocreatine (PCr) recovery reflects the oxidative ATP synthesis rate and was shown to be correlated with in vitro measurements of oxidative capacity (McCully et al. 1993). Briefly, the exercise protocol consisted of dynamic knee extensions against a rubber band (supine position, 1 extension/s, different resistance levels adapted to each subject's strength). Default exercise duration was 28s and ^31^P MR spectra were obtained before, during, and for 9 min after the end of exercise from the quadriceps. The recovery of PCr was fitted to the following formula PCr (t) = PCr_0_ + ΔPCr (1 – e^−k*t^), with PCr_0_ as the PCr signal intensity at the beginning of recovery, and ΔPCr as the exercise-induced decrease of the PCr signal. The oxidative phosphorylation capacity (ATP_max_) was computed as the product of the PCr recovery rate constant (k) and the resting PCr content obtained from the resting spectrum and assuming a constant ATP concentration of 8.2 mmol/L (Conley et al. 2000). This method is described in detail in a previous manuscript (Broskey et al. 2014).

### Exercise efficiency computations

Gross efficiency (GE) was computed as the power output (watts converted in kcal/min) over exercise energy expenditure (EEE in kcal/min) during the 1-h submaximal bike test and expressed as a ratio (Eq. 1) as previously described (Amati et al. 2008). Mean values of work, 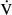
O_2_ and 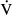
CO_2_ were averaged during the last 3 min at times 30, 45, and 60 min. The first 15 min of data were, if needed, used for workload adjustments to match exactly 55% of 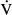
O_2peak_ and thus were discarded. The reliability (test–retest) of this method and the absence of learning effect has been demonstrated in a previous manuscript (Amati et al. 2008).



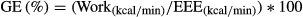
1

Net efficiency (NE) was measured from the submaximal test as power output (watts converted in kcal/min) over EEE minus REE (Eq. 2). It is important to note that due to research settings limitations, REE was measured on a different day as described above, and we did not measure REE in the seated position on the ergometer nor were any measurements of unloaded pedaling taken for the baseline subtraction for all volunteers.



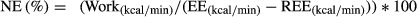
2

Delta efficiency (DE) was measured in two ways. First, DE was obtained as the change in power output (delta watts converted in kcal/min) over the change in EEE (delta EEE) between two stages of the GXT (Eq. 3). Due to the fact that we witness some extremes, on one side 2 athletes did 6–8 stages, while on the other side of the spectrum one sedentary subject did only three stages; the two time points that were always present for all subjects and that were used for this computation were the peak and stage 1. Mean values of 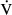
O_2_ and 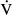
CO_2_ of the last 30 sec of these two stages were used for this computation. Secondly, 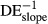 was also obtained by plotting a regression line for each subject with power output on the *X* axis and oxygen uptake on the *Y* axis. The inverse of the slope is DE (expressed in % by multiplying it *100) as described originally by Gaesser and Brooks (1975) and used in other publications (Sidossis et al. 1992; Marsh et al. 2000). Further, this second method was discussed recently by Reger et al. (2013), who stated the usefulness of reporting slopes and intercepts for delta efficiency values; with the former representing metabolic cost of biological processes as work rate increases and the latter being the metabolic cost of biological processes that remain stable at higher work rates. For all of our subjects, we took a minimum of three time points, which was previously reported as the minimum to produce a linear line for estimating DE from a slope (Poole et al. 1992).



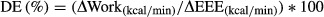
3

For all of these computations, EEE was calculated adapting the formula of Brouwer (Brouwer 1957) as previously described (Amati et al. 2008).

### Statistical procedures

Data are reported as mean ± SEM. Group differences were analyzed by independent T tests. Correlations were performed with Spearman *ρ* correlation coefficient. The significance level was set at 0.05. Statistical analyses were performed using JMP v.9 (SAS, Cary, NC).

## Results

Fifteen athletes (A) and 23 sedentary (S) men and women participated in this study (Table1). Groups did not differ in gender proportions or age. S was significantly higher in weight, BMI, and percent body fat. 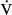
O_2peak_ was greater in A than S when normalized by LBM.

**Table 1:** Subject characteristics.

	Athletes (*n* = 15)	Sedentary (*n* = 23)
Gender, Male/Female	9/6	13/10
Age	68.53 ± 1.19	65.74 ± 0.96
Body Weight (kg)	61.95 ± 3.64	85.08 ± 2.94*
Body Mass Index (kg/m^2^)	21.91 ± 0.99	28.22 ± 0.80*
Percent Body Fat (%)	20.01 ± 1.95	32.16 ± 1.58*
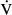 O_2peak_ (L/min)	2.29 ± 0.15	2.11 ± 0.12
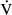 O_2peak_ (mL/min/kg LBM)	47.48 ± 1.75	37.93 ± 1.41*

Values are mean ± SEM, LBM, lean body mass.

* Significant difference between groups, *P *<* *0.05.

**Figure 1:**
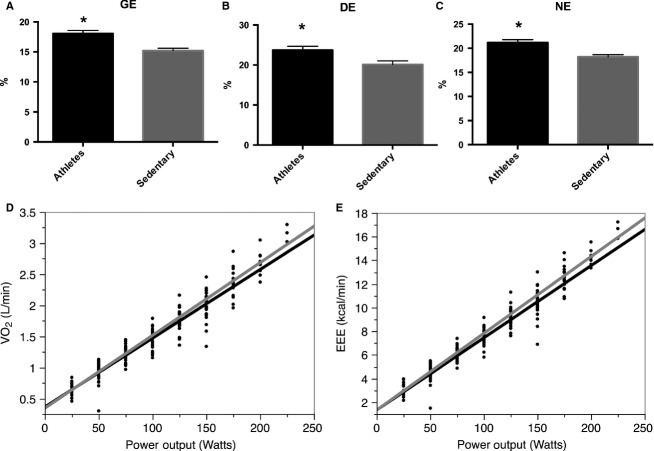
Exercise efficiency differences between athletes and sedentary age-matched older adults. Exercise efficiency is expressed as either gross efficiency (GE, panel A), delta efficiency (DE, panel B), net efficiency (NE, panel C), the inverse of the slope obtained by regression lines of power output over oxygen uptake (VO_2_, panel D), or the slope between exercise energy expenditure (EEE) over power output (panel E). *P < 0.05.

For GE, we fixed the 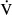
O_2_ during the last three stages of the submaximal bike test, the average 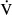
O_2_ was at 1.25 ± 0.07 L/min for the athletes and 1.23 ± 0.09 L/min with no statistical difference between groups (*P* = 0.92). These values, which corresponded to 55% of each subjects' 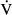
O_2peak_, produced the following power outputs for A (77.27 ± 6.08 W) and S (66.62 ± 5.02 W). There were no statistical differences between these power outputs (*P* = 0.19). The 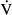
O_2_ corresponding to this wattage was checked to be below the ventilatory threshold; thus, removing any possibility of a slow component during this moderate intensity exercise test. As shown in Figure1, A had both a higher GE and DE than S (panel A and B). Mean regression lines for each group are presented in panel D where Mean 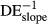 for A was 10.31 ± 0.44% and 9.97 ± 0.35% for S (*P *=* *0.54). We also look at the slopes using EEE instead of 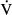
O_2peak_ obtaining similarly nonsignificant results between A and S (panel E).

Resting energy expenditure was higher in S than A with 0.97 ± 0.04 kcal/min versus 0.86 ± 0.03 kcal/min, respectively (*P *<* *0.05). EEE was similar at 55% of 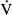
O_2peak_ with 6.04 ± 0.38 kcal/min in the S and 6.21 ± 0.46 in the A (*P *>* *0.05). This gave a NE higher in the A compared to the S (Fig.1, panel C). Although REE is to be taken with caution as it was performed on a different day that the submaximal exercise test, this does not take away the message of the sedentary being less efficient. This is the nature of the different efficiencies and the importance of presenting their various expressions.

**Figure 2:**
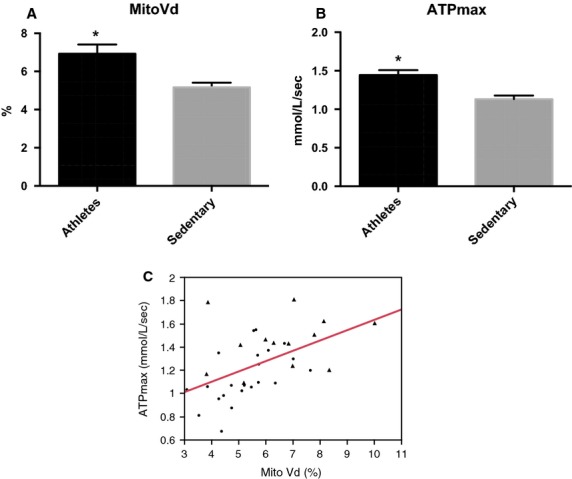
Mitochondrial content and function differences between athletes and sedentary age-matched older adults. Comparison between mitochondrial volume density (MitoVd, panel A) and ATP_max_ (panel B), relationship between MitoVd and ATP_max_ (panel C). *P < 0.05. Athletes (triangle) and sedentary (circle) groups.

With regard to mitochondrial content and function (Fig.2), A had a higher MitoVd (panel A) and higher ATP_max_ (panel B) than S. Confirming our results of the previous study (Broskey et al. 2014), these two outcomes are positively related (*ρ = *0.57, *P *=* *0.0002, panel C).

**Figure 3:**
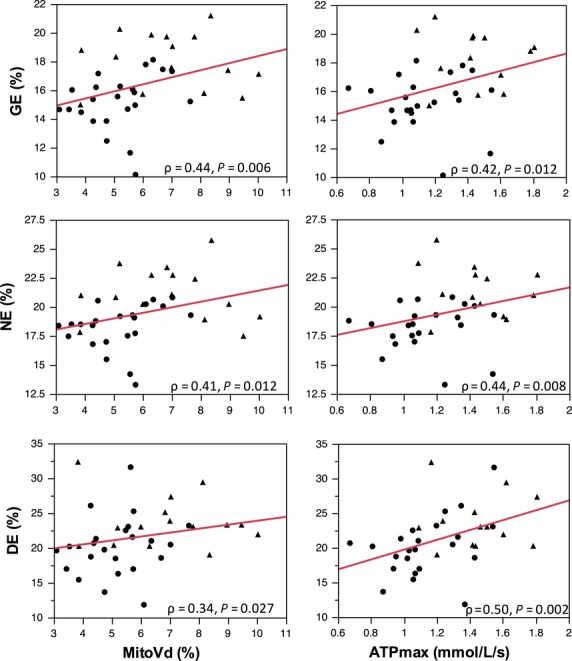
Relationship between exercise efficiencies (GE, gross efficiency; NE, net efficiency; DE, delta efficiency) and mitochondrial content (MitoVd) and function (ATPmax). Athletes (triangle) and sedentary (circle) groups. *ρ* = spearman rho correlation coefficient.

Spearman correlations between the exercise efficiencies and mitochondrial measurements are presented in Table2. Strong correlations exist between both mitochondrial content (MitoVd) and function (ATP_max_) with GE, NE, and DE. The plots of these correlations are shown in Figure3.

**Table 2:** Spearman correlations between exercise and mitochondrial measurements.

	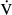 O_2peak_	GE	NE	DE	ATP_max_	MitoVd
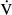 O_2peak_ (mL/min/kg LBM)	1.0	0.69**	0.53*	−0.12	0.19	0.43*
GE (%)		1.0	0.95**	0.10	0.50*	0.36*
NE (%)			1.0	0.13	0.42*	0.44*
DE (%)				1.0	0.44*	0.41*
ATP_max_ [mmol/L/s]					1.0	0.60**
MitoVd (%)						1.0

GE, gross efficiency; NE, net efficiency; DE, delta efficiency; ATP_max_, maximal rate of ATP production; MitoVd, mitochondrial volume density.

* *P *<* *0.05

** *P *<* *0.0001.

## Discussion

Exercise efficiency is higher in young individuals that are endurance trained than those who live a sedentary lifestyle (Mogensen et al. 2006). Furthermore, efficiency is lower in older adults than younger individuals (Martin et al. 1992; Malatesta et al. 2003; Mian et al. 2006; Ortega and Farley 2007; Conley et al. 2013b). Here we show that exercise efficiency is also higher in older athletes than those who are sedentary and matched for age. In addition, we show that GE, NE, and DE correlate with both mitochondrial content expressed as mitochondrial volume density and mitochondrial function expressed as in vivo maximal ATP production.

Our finding that exercise efficiency is greater in those who engage in physical activity is consistent with previous findings in younger subjects (Boning et al. 1984; Mogensen et al. 2006). In our cohort, the athletes expended less energy for the same relative power output. Therefore, they were more efficient, with higher percentages than the sedentary group of all efficiency measurements. Thus, our data shows that athletes have a higher efficiency in an elderly age-matched population similar to younger athletes versus younger sedentary counterparts (Mogensen et al. 2006). Further, the efficiency values found in our older athletes are in the range with the theoretical values published in 1969 by Whipp and Wasserman (1969). Using a cohort of 8 young males (mean age 25 ± 2.3), the authors concluded that since the energy release for phosphorylative coupling is around 60% and the energy required for contractile coupling being around 53%, the product of these two for muscular efficiency should give a maximal value of approximately 32%.

Part of our observations may be explained by fiber type content. We have shown previously that older athletes have significantly more type I muscle fibers than age-matched sedentary individuals (Amati et al. 2011). It has been shown that individuals with more type 1 fibers display higher levels of cycling efficiency (Coyle et al. 1992). On the contrary, differences in efficiencies between groups cannot be explained by the athletes being better at cycling since most of our athletes engaged in running rather than cycling endurance activities. In further support of this claim, researchers have reported in the past no differences in exercise efficiency between well-trained cyclists and recreational cyclists (Boning et al. 1984; Nickleberry and Brooks 1996). It could also be thought that athletes would have more of a preferred biking cadence than the sedentary group, which could affect efficiency. However, this is not the case in our cohort since we controlled the pedaling cadence for both groups during the test (mean cadence for A 67 ± 1 vs. S 65 ± 1, *P* > 0.05). The fact that 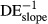 and other outcomes from the regression analyses of the GXT were not significantly different between our cohorts may be explained by the limitations of such computations in a population made of extreme characteristics. Practically, as some of the sedentary women reached 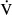
O_2peak_ after three stages, we first computed all slopes in all subjects with all time points and repeated the procedure with only submaximal time points, reaching similar results. We believe that the variability of these results reflects the reality of the population between 60 and 80 years old. Thus, in this broad population, we believe that ‘static’ computations of efficiency (GE and NE), such as using 1 h of submaximal exercise with clear evidence of steady state, are more accurate than ‘dynamic’ measures taking into account each step of the GXT where the number of stages is highly variable.

In addition, the athletes group had both a higher mitochondrial content and function, which correlated positively with all efficiencies. Therefore, although causality cannot be proved in this study, the higher efficiency in the athletes group could be due to both a higher mitochondrial content and function. Two studies in the past showed no relationship with citrate synthase activity (typically used a marker of mitochondrial content/function) and exercise efficiency (Mallory et al. 2002; Mogensen et al. 2006). Citrate synthase measurements have a downside in that it only represents one enzyme of the TCA cycle. It is difficult to actually decipher how much it portrays mitochondrial content and/or function. Instead in our manuscript, we have separate measures for mitochondrial content and function and show a relationship with all types of exercise efficiency.

In regard to age, several cross-sectional studies have shown that older adults expend more energy than younger counterparts of the same activity levels (Martin et al. 1992; Malatesta et al. 2003; Mian et al. 2006; Ortega and Farley 2007). More recently, Conley et al. (2013b) showed that a reduction in delta efficiency in older adults, compared to younger, is due to a reduction in mitochondrial efficiency. In that manuscript, Conley normalizes ATP_max_ by mitochondrial content and defines this as “mitochondrial efficiency”. Although our objective was not to compare different age groups, but to compare athletes versus sedentary older subjects, we explored our data using Conley's approach. Upon normalizing ATP_max_ with mitochondrial volume, our values of mitochondrial efficiency were similar to the elderly group in Conley's paper (approximately 0.2 for both studies). However, in our cohort, we see no differences between athletes and sedentary groups in regard to mitochondrial efficiency using this normalization and no relationship with any measure of exercise efficiency. These differences in mitochondrial efficiency between the two studies could be explained by the different exercise protocols used for the measurement of delta efficiency. In their paper, they used a ramp protocol with workloads increasing on a fixed rate of 10, 12 or 14 W/min, based on self-reported fitness and body mass.

In summary, older adults who are regularly participating in structured physical activity have higher exercise efficiency than age-matched sedentary counterparts. This is also true for both mitochondrial content and function. Elderly who are more athletic can exercise more efficiently and utilize a lesser amount of energy for a given power output; thus, conserving energy stores and exerting less effort. The higher exercise efficiency in the athletes group can be explained by a higher amount of mitochondria occupying their skeletal muscle volume as well as a faster ability to regenerate ATP. Therefore, continuous exercise at an older age can possibly ameliorate the conditions of disease states attributed to decreases in mitochondrial function by increasing mitochondrial content, function, and whole-body efficiency.
